# Multifaceted Roles of ALG-2 in Ca^2+^-Regulated Membrane Trafficking

**DOI:** 10.3390/ijms17091401

**Published:** 2016-08-26

**Authors:** Masatoshi Maki, Terunao Takahara, Hideki Shibata

**Affiliations:** Department of Applied Molecular Biosciences, Graduate School of Bioagricultural Sciences, Nagoya University, Furo-cho, Chikusa-ku, Nagoya 464-8601, Japan; takahara@agr.nagoya-u.ac.jp (T.T.); shibabou@agr.nagoya-u.ac.jp (H.S.)

**Keywords:** ALG-2, ALIX, calcium, COPII, ESCRT, membrane repair, multivesicular body, PDCD6, protein–protein interaction, Sec31A

## Abstract

ALG-2 (gene name: PDCD6) is a penta-EF-hand Ca^2+^-binding protein and interacts with a variety of proteins in a Ca^2+^-dependent fashion. ALG-2 recognizes different types of identified motifs in Pro-rich regions by using different hydrophobic pockets, but other unknown modes of binding are also used for non-Pro-rich proteins. Most ALG-2-interacting proteins associate directly or indirectly with the plasma membrane or organelle membranes involving the endosomal sorting complex required for transport (ESCRT) system, coat protein complex II (COPII)-dependent ER-to-Golgi vesicular transport, and signal transduction from membrane receptors to downstream players. Binding of ALG-2 to targets may induce conformational change of the proteins. The ALG-2 dimer may also function as a Ca^2+^-dependent adaptor to bridge different partners and connect the subnetwork of interacting proteins.

## 1. Introduction

Two decades have passed since ALG-2 was first reported as a calcium-binding pro-apoptotic factor of mouse T cells encoded by the *Apoptosis-linked gene 2* [1]. Since ALG-2 (also named PDCD6) has no catalytic activity, physiological functions of ALG-2 are exerted by Ca^2+^-dependent interaction with its target proteins. Discovery of ALIX (ALG-2-interacting protein X)/AIP1 (gene name, PDCD6IP) as the first ALG-2-interacting protein linked ALG-2 and the cell death pathway [2,3]. Although *ALG-2* gene knockout mice behaved normally and showed no abnormalities in the immune system [4], accumulating evidence suggests that ALG-2 is involved in regulation of cell death, cell division, and signal transduction in cultured cells as reviewed in the past [5,6]. After discovery of ALIX as an accessory factor in the ESCRT (endosomal sorting complex required for transport) system [7,8,9,10] and identification of novel ALG-2-interacting proteins in different systems, much attention has been paid in recent years to the role of ALG-2 in membrane repair and vesicular transport [11,12]. In this review, we focus on recent findings of the structural basis of target recognition and endowed diverse functions of ALG-2, particularly unveiled from interacting proteins, in the membrane trafficking.

## 2. Structure of ALG-2

**Figure 1:**
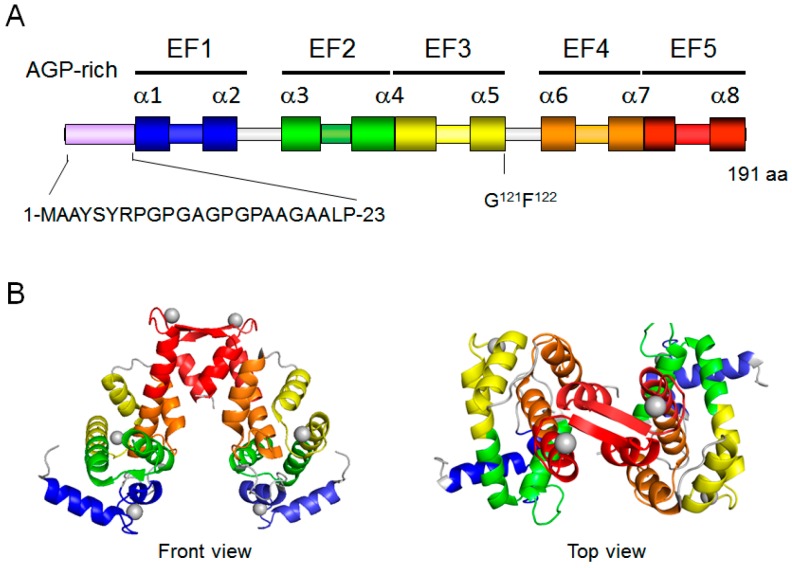
Structure of human ALG-2. (**A**) The N-terminal region is rich in Ala/Gly/Pro. A penta-EF-hand (PEF) domain has five EF hands (EF1–EF5) with eight α-helices (α1–α8). An alternatively spliced isoform lacks two residues (Gly^121^Phe^122^); (**B**) X-ray crystal structure of the dimeric Ca^2+^-bound form of human ALG-2 (PDB code: 2ZN9) is presented by cartoon using PyMol. EF-hands are shown in different colors for each EF-hand module corresponding to EF1–EF5 in panel **A**. Gray spheres, calcium atoms.

Human ALG-2 (22 kDa, 191 amino acids) has an Ala/Gly/Pro/-rich N-terminal tail followed by a penta-EF-hand (PEF) domain (Figure 1), which is a region comprised of approximately 170 amino acid residues containing five repeated helix-loop-helix Ca^2+^-binding motifs (EF-hands) [13]. The PEF family includes ALG-2, peflin, sorcin, grancalcin, and calpain subfamily members in mammals [14]. Generally, a pair of EF-hands forms a discrete domain in Ca^2+^-binding proteins with even numbers of EF-hands [15]. Stabilization of the paired EF-hands is facilitated by hydrophobic interactions within two types of motifs (cluster I and cluster II), each comprised of three residues separately residing at the pairing partner EF-hands [16]. PEF proteins have a unique feature of utilizing the isolated fifth EF-hand (EF5) to pair with that of another PEF protein molecule to form a homodimer or heterodimer: ALG-2/ALG-2 [3,17] and ALG-2/peflin [18], sorcin/sorcin [19], grancalcin/grancalcin [20] and sorcin/grancalcin [21], and large and small subunits of typical calpains [22]. An alternatively spliced isoform lacking Gly^121^Phe^122^ is generated by utilization of an upstream splicing donor site in mouse and human pre-mRNAs [23,24] and is designated ALG-2^ΔGF122^ in this review article.

## 3. Ca^2+^-Binding Capacity

Two high affinity sites with a dissociation constant (*Kd*) in a μM order (1.2 μM) and one lower affinity site (300 μM) were estimated for Ca^2+^-binding capacity of ALG-2 by flow dialysis in the presence of 0.5% Tween (a 5–10-fold lower affinity in the absence of the detergent) [23,25] and isothermal titration calorimetry [26]. An alternatively spliced isoform, ALG-2^ΔGF122^, shows a lower Ca^2+^-binding affinity. Substitutions of amino acids essential for metal coordination in both EF1 and EF3 (E47A/E114A) abolished a Ca^2+^-induced conformational change detected by circular dichroism (CD) [17]. Typical EF-hand Ca^2+^ coordination at EF1 and EF3 was verified by X-ray crystal structural analyses of mouse and human ALG-2, and an additional calcium atom was observed at EF5 with incomplete coordination, suggesting a lower affinity site [27,28]. Interestingly, a high affinity Mg^2+^-binding site (*Kd* ~ 20 μM) was predicted in EF5, which would be fully occupied under the condition of a physiological concentration of Mg^2+^ in the cell (~mM) [26].

## 4. Interacting Proteins

Comparison of the 3D structures of Ca^2+^-free and Ca^2+^-bound forms of ALG-2 revealed only a small change in the overall structure [28]. However, results of fluorospectrometric analysis of recombinant proteins indicated that conformational change and exposure of hydrophobic patches occur in a Ca^2+^-dependent manner at μM order concentrations of Ca^2+^, suggesting that ALG-2 functions as a Ca^2+^ sensor [17,29]. A search for ALG-2-interacting proteins is one of first approaches to reveal physiological functions of ALG-2. Table 1 summarizes published mammalian ALG-2-interacting proteins, UniProt IDs represented by human proteins, approximate binding regions, binding ability to the alternatively spliced isoform (ALG-2^ΔGF122^), binding motifs, employed methods, functions, and references describing interactions [2,3,25,28,30,31,32,33,34,35,36,37,38,39,40,41,42,43,44,45,46,47,48,49,50,51,52,53,54,55,56]. A variety of proteins have been identified by several independent groups employing combinations of different methods for validation: (i) the yeast two-hybrid (Y2H) method; (ii) Western blotting or mass spectrometry of recovered protein samples after immunoprecipitation (IP) with specific antibodies; (iii) pulldown (PD) with glutathione-*S*-transferase (GST)-fused proteins or with Strep-tagged proteins; (iv) Far Western (FW) blotting; and (v) in vitro binding of recombinant proteins with a surface plasmon resonance (SPR) biosensor. A database homology search with a query sequence of previously determined interacting motifs is also useful for the first screen. A report of Fas as an ALG-2-binding protein [47] is controversial due to a later revealed erroneous reaction of the antibody used [57]. Results of the Y2H method alone are not included in the list due to low reliability. Interacting proteins suggested by results obtained by using any single method need further validation for specific binding including identification of a binding sequence. Interactions only published by one group without such validation have to be taken with caution as well.

Co-immunoprecipitation and pulldown assays using cell lysates do not necessarily indicate direct interaction. FW blotting (also called overlay assay) with biotin-labeled or epitope-tagged recombinant ALG-2 is used for detection of direct binding of unpurified target proteins after SDS-polyacrylamide gel electrophoresis (PAGE) separation.

## 5. Mode of Binding

### 5.1. Binding Pockets in ALG-2

**Figure 2:**
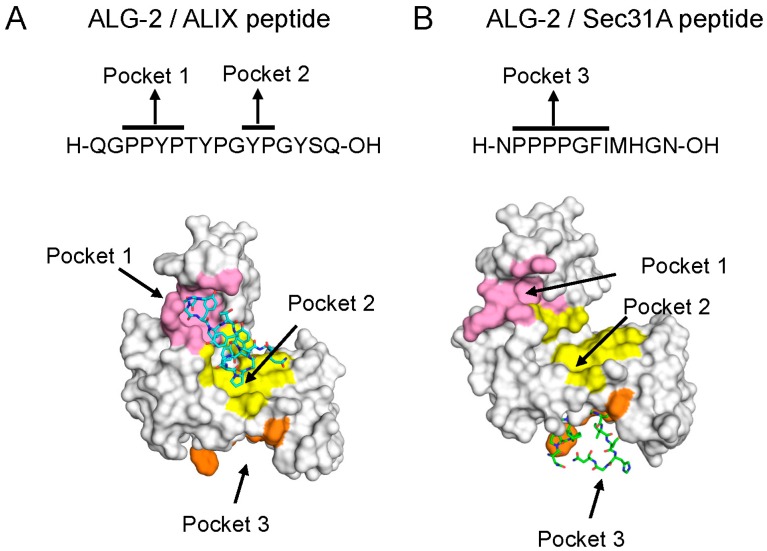
Different hydrophobic pockets used for the binding of ALG-2 to ALIX (ALG-2-interacting protein X) and Sec31A: (**A**) complex between ALG-2 and the ALIX peptide (PDB code, 2ZNE, chains A and C); and (**B**) complex between ALG-2 and the Sec31A peptide (PDB code, 3WXA, chains A and C). Peptides are shown in a stick model. Figures were taken from Ref. [58] and modified.

ALG-2-interacting proteins are classified into two major groups by structural features: (i) proteins containing binding sites in Pro-rich regions (PRRs) and (ii) proteins containing no obvious PRRs. By successfully narrowing down the binding sites in ALIX, PLSCR3 (phospholipid scramblase 3), and Sec31A, two different binding motifs were predicted [30,31,40,41]. Synthetic oligopeptides were used for X-ray crystallographic analyses of the ALG-2/oligopeptide complexes for ALIX and Sec31A in the presence of Zn^2+^ in place of Ca^2+^ [28,58]. While the ALIX peptide binds ALG-2 at two adjacent hydrophobic pockets largely formed by residues from EF3 to EF5 as well as by Y180 (EF5) of a dimerizing molecule (Pocket 1, PPYP) and from EF2 to EF4 (Pocket 2, YP), the Sec31A peptide binds at a different pocket largely formed by residues from EF1 to EF3 (Pocket 3, PPPPGFI) (Figure 2).

### 5.2. Mechanism of Binding

Upon binding to Ca^2+^, the classic EF-hand protein, calmodulin, changes its conformation from a “closed” state to “open” state and a further gross change makes the two lobes (N-terminal half, EF1 and EF2; C-terminal half, EF3 and EF4) grab a target peptide [59]. In contrast, upon metal (Ca^2+^ or Zn^2+^) binding to EF3, ALG-2 exhibits only a small shift of α-helix 5 that leads to a change in configuration of the R125 side chain, resulting in a sufficient move to make Pocket 1 accessible to the critical PPYP motif of ALIX [28]. Since there are no significant changes in Pocket 3 structures among metal-free, metal-bound and Sec31A peptide-bound states, dynamic structural differences not revealed by crystal analysis may account for Ca^2+^-dependent activation of Pocket 3 [58]. Inability of the alternatively spliced isoform (ALG-2^ΔGF122^) to bind to ALIX and to a subset of other proteins (Table 1) is not caused by loss of the aromatic residue (F122), but deletion of the two residues shortens α-helix 5 and changes the configuration of the R125 side chain so that it partially blocks Pocket 1 [60]. Unexpectedly, substitution of F122 with Ala increased the ALIX-binding capacity [60]. ALG-2^ΔGF122^ maintains the binding capacity to Sec31A, PLSCR3 and PATL1 (protein associated with topoisomerase II (PAT)-like 1) [41,54]. 

### 5.3. ALG-2-Binding Motifs (ABMs)

**Figure 3:**
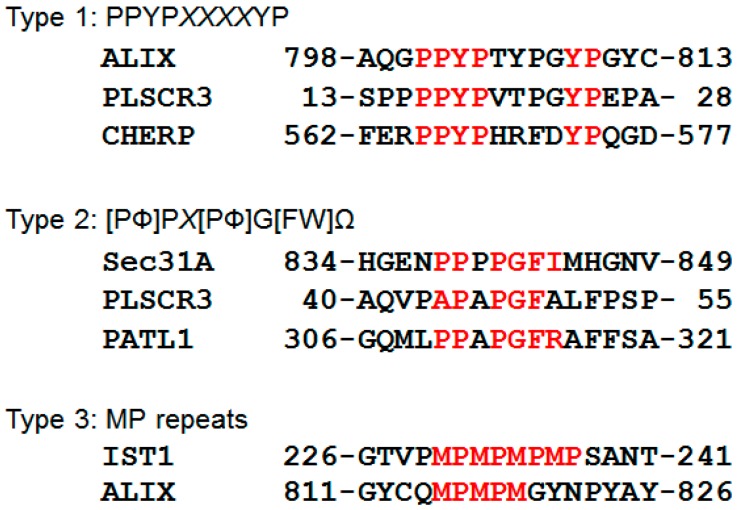
Three types of Pro-rich ALG-2-binding motifs. Amino acid sequences of human Pro-rich proteins containing ALG-2-binding motifs (Type 1, Type 2, and Type 3) are presented. *X*, any amino acid; Φ, hydrophobic; Ω, large side chain. Amino acids compatible with ALG-2-binding motifs are indicated in red except for *X* positions. IST1, increased sodium tolerance-1; PLSCR3, phospholipid scramblase 3; PATL1, protein associated with topoisomerase II (PAT)-like 1; CHERP; calcium homeostasis and endoplasmic reticulum protein.

Determination of ALG-2-binding sites in the interacting proteins and amino acid substitution experiments have revealed three types of Pro-based ALG-2-binding motifs (ABMs) (Figure 3) and one non-Pro-based ABM.

#### 5.3.1. Type 1

Type 1 motif (ABM-1), represented by ALIX, is comprised of two sub-core sequences (PPYP and YP, separated by 4 residues) and is found in the confirmed minimal binding sequences of PLSCR3 and CHERP. Regardless of reduction in binding affinity, PPYP is substitutable with PYP and P*X*YP (*X*, variable) [28,31]. Although no experimental evidence has yet been obtained, the distance between PPYP and YP may be variable depending on the interval sequence (TSG101 (tumor susceptibility gene 101), 188-CPYPPGGPYP; AnxA11, 4-PGYPPPPGGYP; AnxA7, 4-PGYPPPPGGYP; Scotin, 189-APYPMQYPPPYP). Most ALG-2-interacting proteins that are incapable of binding to ALG-2^ΔGF122^ contain Type I or Type 1-like motifs. VPS37B, VPS37C and TSG101 also contain PPYP, PYP or P*X*YP sequences. Unidentified residues substituting the second core-sequence (YP) may potentiate ALG-2 binding.

#### 5.3.2. Type 2

Type 2 motif (ABM-2), first determined in the Pro-rich region of PLSCR3 and designated P*X*PGF (*X*, any amino acid) [41], was newly defined (Figure 3) on the basis of results of mutational analysis of the Sec31A sequence [58]. The Type 2 motif of PLSCR3 is not optimal (small amino acid, Ala, at the position of Ω following Phe), but downstream residues probably with large side chains may compensate the weak binding ability [41,58]. Since PLSCR3 contains both Type 1 and Type 2 motifs, it retained the capacity to bind to ALG-2^ΔGF122^, but a type 2 deletion mutant did not bind to ALG-2^ΔGF122^ [41].

#### 5.3.3. Type 3

Increased sodium tolerance-1 (IST1) has an MP-repeat (four times, or five times in a polymorphic isoform) sequence in the Pro-rich region. Although this sequence (Type 3, designated ABM-3) alone was not sufficient for ALG-2 binding, it was necessary for IST1 to bind ALG-2 efficiently [36]. IST1 did not bind ALG-2^ΔGF122^. Interestingly, the Type 1 motif in ALIX is followed by a Type 3-like sequence, which may potentiate ALG-2 binding. In experimental immunochemistry, epitope-tagging of a concerned protein with plural epitope sequences is frequently employed to increase the binding affinity (e.g., 3 × FLAG). Similarly, the presence of multiple motifs of the same or different types may contribute to augmentation of binding capacities by compensating with multi-valent suboptimal sequences. 

#### 5.3.4. Non-Pro-Based Motif

ALG-2 also binds non-Pro-rich proteins that do not share apparently similar sequences. The ABH motif (clusters of acidic/basic/hydrophobic residues: 37-EEEDLRRRLKYFF-49) of Mucolipin 1 was essential for the binding, but sufficiency was not demonstrated [45]. Since the binding ability of ALG-2^ΔGF122^ was significantly reduced, Mucolipin 1 may bind ALG-2 in a mode similar to that of Type 1 motif proteins. Since ASK1 (apoptosis signal-regulating kinase 1) binds ALG-2 both in the presence and absence of Ca^2+^ [52], the mode of binding should be completely different from that of other interacting proteins. 

## 6. Interacting Protein Network of ALG-2 in Membrane Trafficking

**Figure 4:**
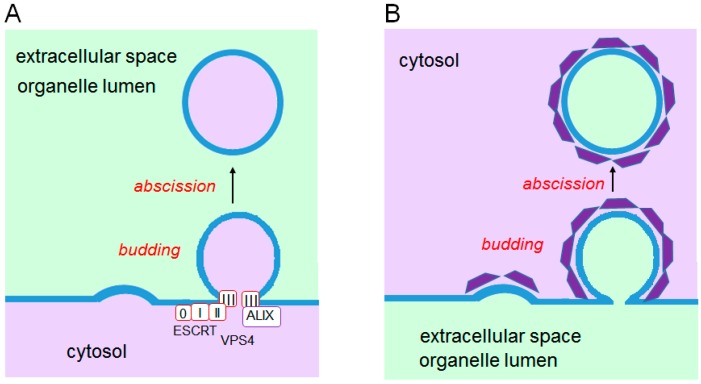
Topologically opposite budding of vesicles. Cytosolic proteins are recruited to the budding sites, but orientations of membrane deformation are opposite. (**A**) Endosomal sorting complex required for transport (ESCRT)-associated vesicle budding occurs from the cytosolic face of the membrane towards the extracellular space or into the organelle lumen; (**B**) Membrane coated with coat proteins (clathrin, coat protein complex I (COPI), and COPII) buds from the plasma membrane or organelle membranes into the cytosolic space.

Membrane trafficking is mediated by vesicles that are carried from originating membranes to target membranes. Biogenesis of vesicles is initiated by deformation of the lipid bilayer in a restricted membrane area and then by budding and finally by abscission. Budding occurs in topologically opposite directions: (i) budding away from the cytosol; and (ii) budding into the cytosol (Figure 4). ALG-2 participates in part and plays regulatory roles by Ca^2+^-dependently interacting with key players in both systems.

### 6.1. ESCRT System

**Figure 5:**
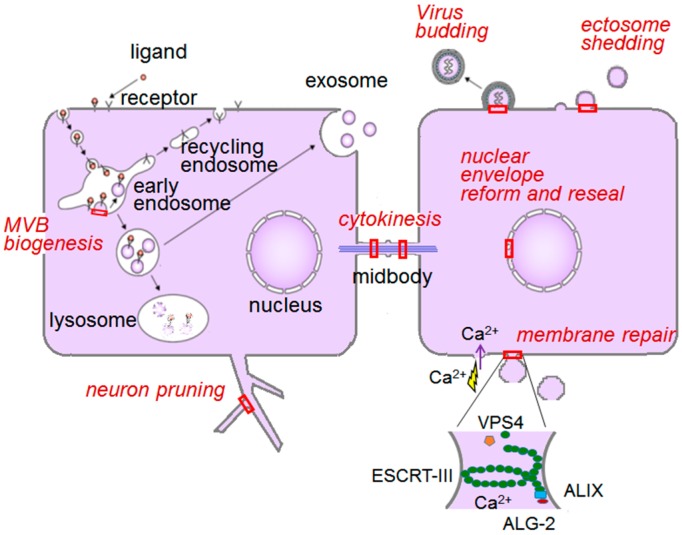
ESCRT system in mammalian cells. ESCRT-associated membrane deformation and abscission machinery works in a versatile phenomenon at particular sites in the cell as indicated by red boxes. VPS, vacuolar protein sorting; MVB, multivesicular body.

Endocytosed ubiquitinated membrane proteins, which are destined for lysosomal degradation, are recruited to a specialized area on endosomal membranes by multiprotein complexes named ESCRT-0, -I and -II. Sequentially transferred cargoes are further transported into inward budding vesicles by concomitant membrane deformation with spirally polymeric ESCRT-III [10]. Disassembly of the ESCRT complexes by the AAA-type ATPase VPS4 pinches off the buds into multivesicular bodies (MVBs). This ESCRT system in MVB sorting is conserved from yeast to humans [10], but remodeled machineries consisting of ESCRT-III/VPS4 as well as parts of the complexes and accessories are used in different combinations for exerting specific functions, such as virus budding, cytokinetic final abscission of daughter cells at the midbody, extracellular vesicle release, plasma membrane repair, neuron pruning, nuclear pore complex (NPC) surveillance, and nuclear envelope (NE) reformation (Figure 5) (see Refs. [10,11] for reviews and references therein). ALG-2 has been shown to be involved only in the following two ESCRT systems, but involvement in other systems is not necessarily excluded.

#### 6.1.1. Endosomal Sorting Pathway

**Figure 6:**
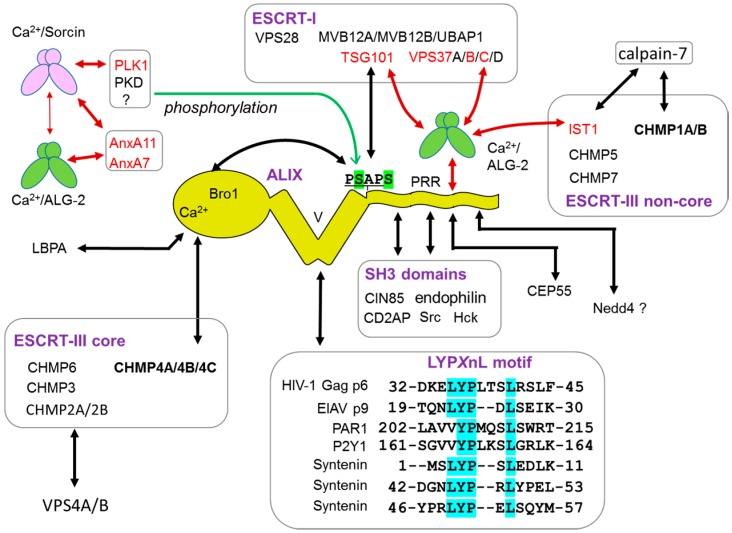
Subnetwork of ALG-2 and interacting proteins in the ESCRT system. Physical links are indicated by double-headed arrows colored in red for PEF proteins and in black for non-PEF proteins and lysobisphosphatidic acid (LBPA), a phospholipid enriched in MVB. Sites of phosphorylation by cytokinetic kinases are highlighted in green, and the TSG101 (tumor susceptibility gene 101) docking site is underlined in ALIX. Sequences of proteins containing the LYP*X*nL-motif and interacting with the V domain of ALIX are highlighted in cyan at the conserved residues. PRR, Pro-rich region. CEP55, centrosomal protein of 55 kDa; PLK1, Polo-like kinase 1; PKD, protein kinase D. The mode of interaction between ALG-2 and Sorcin is different from between ALG-2 and Ca^2+^-dependent targets (see Text).

The ALG-2-interacting protein X, ALIX, has three distinct domains: N-terminal Bro1 domain, middle V domain and C-terminal Pro-rich region (PRR) (Figure 6). CHMP4 (ESCRT-III core subunit) and TSG101 (ESCRT-I subunit) bind ALIX at the Bro1 domain and the PRR, respectively. The V domain binds the so-called late domain of HIV (human immunodeficiency virus)-1 p6 Gag and EIAV (equine infectious anaemia virus) Gag p9 by recognizing the LYP*X*nL motif (Figure 6) [7,8,61]. Thus, ALIX promotes budding of retrovirus particles by bridging ESCRT-I and ESCRT-III. The V domain plays roles in cargo recognition by binding to the YP*X*nL motif of G protein-coupled receptor PAR1 and P2Y1 for ubiquitin-independent MVB sorting [62,63]. Binding of the ALIX V domain to polyubiquitin has also been reported [64]. 

By using conformation-specific monoclonal antibodies of ALIX for co-immunoprecipitation and analysis of interacting abilities of mutants, ALIX has been shown to exhibit inactive and active conformations [65]. The closed conformation is maintained by intramolecular interaction between hydrophobic Patch 2 of the Bro1 domain and the TSG101 docking site (717-PSAP motif) in the PRR. Relief of intramolecular interaction is achieved by mutations of interacting residues [65], binding of ALG-2 in a Ca^2+^-dependent signaling pathway [66], and phosphorylation of Ser at 718-SAPS-720 by kinases that are activated in the mitotic phase (PLK1 (Polo-like kinase 1), PKD (protein kinase D), and others) [67]. These actions induce global conformational change from an auto-inhibitory closed form to an open active form that permits interaction with partner proteins at hydrophobic Patch 1 for CHMP4 in the Bro1 domain and V domain for EIAV p9 or ubiquitinated epidermal growth factor receptor (EGFR) [68]. Knockdown of ALIX or ALG-2 with siRNAs suppressed MVB sorting of the ligand-bound EGFR, promoted sustained activation of ERK1/2 and retarded EGFR degradation [66,68]. While S718-S721 phosphorylation of ALIX was not involved in the regulation of MVB sorting of endocytosed EGFR, it was required for ALIX to function in cytokinetic abscission and retroviral budding [67]. On the other hand, ALG-2 was not required for the final abscission in cytokinesis or for retroviral budding. 

Knockdown effects of ALIX and ALG-2 on MVB sorting of EGFR reported by Kuang’s group [66,68] challenge the notion that ALIX is not critically involved in MVB sorting of ubiquitinated EGFR and that His domain-containing protein tyrosine phosphatase (HD-PTP), a paralog of ALIX, is utilized for MVB sorting as a functional mammalian ortholog of yeast Bro1 [69,70]. Using mouse embryonic fibroblast (MEF) *Alix*^−/−^ cells, Mercier et al. [71] demonstrated that ALIX regulated fluid-phase endocytosis and internalization of cargoes, including interleukin-2 receptor (IL2R) β chain and cholera toxin B (CTxB), via clathrin-independent endocytosis (CIE) but had no apparent effects on clathrin-mediated endocytosis (CME) of the transferrin receptor or downstream endosomal trafficking. Degradation of EGFR was delayed in *Alix*^−/−^ cells at a high EGF concentration (100 ng/mL, condition for CIE) but not at a low EGF concentration (2 ng/mL, condition for CME). ALIX binds endophilins and other SH3 domain-containing proteins (CIN85/SETA, CD2AP, Src, Hck) [72,73]. Knockdown of endophilin-A suppressed CTxB uptake [71]. Moreover, rescue experiments by expression of ALIX mutants in *Alix*^−/−^ cells indicated that a binding site of endophilins in ALIX was essential for complementation, but those of TSG101 and CIN85 were dispensable. It would be interesting to know whether ALG-2 and Ca^2+^ regulate CIE by inducing conformational change of ALIX.

HD-PTP has the Bro1 domain, the V domain and the PRR as well as a phosphotyrosine phosphatase (PTP) domain and the PEST sequence in addition to the ALIX structure [32]. The HD-PTP Bro1 domain binds not only CHMP4 but also STAM2 (ESCRT-0 subunit), and the V domain binds UBAP1 (ubiquitin-binding ESCRT-I subunit) [32,74,75]. The PRR of HD-PTP binds ALG-2, TSG101 as well as STAM2 [32,74]. Binding partners of ALG-2 in the ESCRT system are not limited to ALIX and HD-PTP. ALG-2 also binds to selective ESCRT-I subunits (TSG101, VPS37B, VPS37C) and ESCRT-III-like IST1 (Table 1). The 717-PSAP motif for TSG101 in ALIX is dispensable for interaction with ESCRT-I by the Ca^2+^-dependent adaptor action of dimeric ALG-2 that bridges ALIX and ESCRT-I [34]. The bridging capacity of ALG-2 seems weak for HD-PTP and ESCRT-I because VPS37A, which is incapable of ALG-2-binding, is selectively used for UBAP1-containing ESCRT-I [76]. Since the PTP domain of HD-PTP is catalytically inactive [77], differential utilization of ALIX or HD-PTP in the endosomal sorting pathway may depend on differences in recruited interacting partners under experimental conditions used. 

The Bro1 domain of ALIX binds lysobisphosphatidic acid (LBPA), enriched in MVB, and regulates biogenesis of intraluminal vesicles (ILV) and back fusion of ILV to endosomal limiting membranes [78,79,80]. Microvesicles are released into the extracellular space from the plasma membrane (ectosomes, shedding vesicles) or by release of ILV by fusion of MVB with the plasma membrane (exosomes) [81]. ALIX and the ESCRT machinery play critical roles in both ectosome secretion and exosome secretion. Syntenin, a cytoplasmic adaptor of syndecan, interacts with ALIX through LYP*X*nL motifs and regulates exosome biogenesis [82].

#### 6.1.2. Plasma Membrane Repair

Injury of the plasma membrane triggered by pore-forming toxins and various physical stresses is a threat to cell survival, and the wounded membrane needs to be rapidly repaired [83]. Entry of extracellular Ca^2+^ is a major and early signal for this process. Two recent reports indicate that the ESCRT system works in plasma membrane repair [84,85]. ALIX and ESCRT-III proteins were locally recruited in a Ca^2+^-dependent manner to laser-beam-wounded sites and mediated the closure and pinching out (shedding) of the wounded plasma membrane portion [84]. The Bro1 domain of ALIX has a tight Ca^2+^-binding site (*Kd* = 467 nM), and binding to LBPA requires Ca^2+^ [79]. Mutations of either the Ca^2+^-binding site or LBPA-binding site impaired accumulation of ALIX at the wound site [84]. However, it remains unclear whether a wound membrane site is enriched in LBPA and ALIX can function as a Ca^2+^ sensor. Identification of ALG-2 as an initiator of the sequential recruitment of ALIX, ESCRT-III, and VPS4 to laser-wounded sites by live cell imaging has partly answered the question [85]. Importantly, knockdown of ALG-2 impaired accumulation of ALIX, but vice versa was not true, i.e., ALG-2 accumulated at the wounded sites in the ALIX-knockdown cells [85]. Thus, a new question comes up: how is ALG-2 recruited to the wounded sites? Although ALG-2 exposes hydrophobic surfaces upon Ca^2+^-binding and is recovered in membrane fractions [29], phospholipid binding under physiological conditions remains unknown. Annexins, Ca^2+^-dependent phospholipid-binding proteins [86], have been shown to play important roles in membrane repair [87]. Interestingly, copines (C2-type Ca^2+^-dependent phospholipid-binding proteins) and annexin A7, both ALG-2-interacting partners (Table 1), are included in the list of proteins of the plasma membrane injury proteome in which cell surface levels were elevated as a result of ionomycin treatment [85]. Future studies are required to determine whether these phospholipid-binding proteins are involved as upstream factors of ALG-2 in the above-described membrane injury model. Remarkably, ESCRT-0 and ESCRT-II were not recruited to the wounded sites. Among ESCRT-I subunits, only TSG101 was enriched in the wounded plasma membrane [85]. Live cell imaging indicates that accumulation of ALG-2 precedes accumulation of TSG101. Taken together, the results suggest that ALG-2 plays roles similar to the roles of ESCRT-0 in MVB biogenesis, HIV-1 Gag in virus budding, and CEP55 in cytokinesis [11,85]. Interestingly, CEP55 interacts with ALIX and TSG101 at the GPP*X*_3_Y motif [88], which overlaps with the ALG-2-binding site (Type 1 motif) in ALIX [28].

### 6.2. ER-to-Golgi Vesicular Transport

Most integral membrane proteins and secretory proteins are synthesized on endoplasmic reticulum (ER) membranes, correctly folded in the ER lumen, incorporated into ER-derived transport vesicles and reach their final destinations via the Golgi apparatus. Cargo protein sorting and formation of vesicles coated with coat protein complex II (COPII) are accomplished at specialized regions of the ER named ER exit sites (ERES) [89,90]. ALG-2 binds Sec31A, a component of the outer layer of COPII, in a Ca^2+^-dependent manner and is recruited to the ERES to stabilize Sec31A [37,38,39,40]. ALG-2 was shown by in vitro assays to participate in the regulation of ER-to-Golgi vesicular transport at two steps: (i) suppression of homotypic fusion of COPII vesicles [91]; and (ii) attenuation of the budding of COPII vesicles using purified COPII proteins and permeabilized cells [92]. Inhibiting COPII budding and suppressing homotypic COPII fusion would lead to a reduction of ER-Golgi intermediate compartment (ERGIC) function and thereby slow down the transport of cargoes. In other words, it would be expected that ALG-2 depletion would increase the trafficking of cargoes. However, the observed effects of ALG-2 depletion by siRNA on the transport of the temperature-sensitive variant of vesicular stomatitis virus envelope glycoprotein (tsO45 VSV-G), a model cargo, described by three research groups including ourselves are not consistent: no effects in HeLa cells (data not shown in Ref. [37]), mild suppressive effects in normal rat kidney (NRK) cells (figure not shown in Ref. [93]), and accelerating effects in HT1080 cells [94]. The discrepancies are probably due to differences in methods, assay conditions, and cell lines used. By using cyclopiazonic acid (CPA) (the reversible inhibitor of the sarcoplasmic/endoplasmic reticulum Ca^2+^-ATPase, SERCA) and Ca^2+^-free medium, Bentley et al. depleted the luminal Ca^2+^ but maintained the cytosolic Ca^2+^ at the basal level and concluded that the source of Ca^2+^ utilized by ALG-2 in the ER-to-Golgi transport regulation was supplied by luminal Ca^2+^ leaking from the ER or vesicles [91,93]. How and on what signaling event Ca^2+^ leaks from the lumen is not known. It remains to be established whether there are specificities in vesicular transport regulation of cargoes other than VSV-G by ALG-2. There might exist cargoes that are more strictly regulated by Ca^2+^/ALG-2. On-demand regulatory transport from the ER to the Golgi involving Ca^2+^-signaling event may be more beneficial to the cells than unregulated transport in order to avoid wasting energy and substances. 

**Figure 7:**
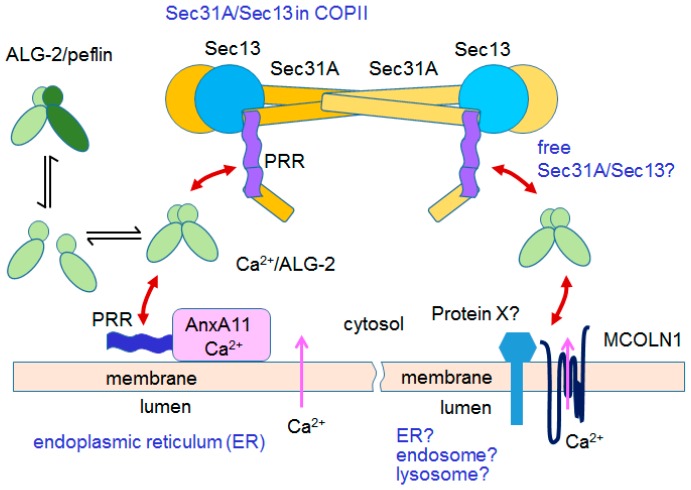
Subnetwork of ALG-2 and interacting proteins in the COPII system. Ca^2+^-dependent physical links with ALG-2 are indicated by double-headed arrows colored in red. Only two pairs of the Sec31A/Sec13 complex in the COPII outer shell are shown for simplicity. Conversion of the ALG-2 dimer and ALG-2/peflin heterodimer may occur in the cell.

A subset of annexin A11 (AnxA11) colocalizes with Sec31A and ALG-2 at the ERES [94]. Physical association of AnxA11 with Sec31A is mediated by dimeric ALG-2 that bridges AnxA11 and Sec31A by functioning as a Ca^2+^-dependent adaptor as in the case of ALIX–ESCRT-I interaction [34,35]. This adaptor function seems specific in selecting partners because ALG-2 did not bridge ALIX and Sec31A [94]. Knockdown of either AnxA11 or ALG-2 accelerated ER-to-Golgi transport of VSV-G in HT1080 cells [94]. AnxA11 may function as a Ca^2+^-dependent membrane anchor to hold the COPII vesicles at the ERES until abscission is ready (Figure 7). It is possible that unknown membrane-tethering proteins associating with ALG-2 also regulate the rate of budding and abscission process. 

### 6.3. Membrane Associated Proteins Interacting with ALG-2

It is rational to presume that ALG-2, working as a Ca^2+^-sensor protein, finds partners more easily in the vicinity of the plasma and organelle membranes through which Ca^2+^ is released into the cytosol. A few integral membrane proteins and membrane-associated proteins are known to interact with ALG-2 in a Ca^2+^-dependent manner: Scotin [46], Mucolipin-1 [45] and PLSCR3 [41].

#### 6.3.1. Scotin

Scotin is a type I transmembrane protein that is localized to the ER membrane and the nuclear envelope [95]. Overexpression of Scotin caused caspase-dependent apoptosis [95]. Interestingly, the promoter regions of both *Scotin* and *ALG-2* genes have p53-responsive elements, and these genes were induced by DNA damage accompanied by transactivation activity of p53, as revealed by luciferase reporter assays [95,96]. The C-terminal cytosolic domain of Scotin was sufficient for the ER localization but the N-terminal luminal cysteine-rich domain was not necessary [95]. The cytosolic domain contains a PRR for interaction with ALG-2 [46], but the relationship between subcellular localization and ALG-2-binding capacity is not known. Scotin is a member of the SHISA family (SHISA1-9) and constitutes a large family of STMC6 (single-transmembrane proteins with conserved 6 cysteines), which is postulated to function as adaptors to regulate other transmembrane proteins [97]. SHISA4 is the closest paralog of Scotin (also named SHISA5). It binds ALG-2 directly as shown by FW blotting with biotin-labeled ALG-2 [54], but examination by other assay methods is required for confirmation of the interaction.

#### 6.3.2. Mucolipin-1

Mucolipin-1 (MCOLN1; TRPML1, transient receptor potential mucolipin 1; non-selective cat ion channel) functions as a vesicular Ca^2+^-release channel in late endosomes/lysosomes and controls membrane trafficking of proteins and lipids by promoting membrane fusion of amphisomes (intermediate organelles of autophagosome/endosome fusion) and lysosomes [98]. Autosomal somatic recessive mutations in the human *MCOLN1* gene cause a lysosomal storage disease, mucolipidosis type IV [99]. Vergarajauregui et al. [45] reported that ALG-2 bound MCOLN1 Ca^2+^-dependently in the region with a cluster of acidic, basic and hydrophobic residues (ABH motif: 37-EEEDLRRRLKYFF-49) present in the N-terminal cytosolic tail (NTail). Although the physiological role of ALG-2 binding to MCOLN1 is not known, aggregation of abnormal endosomes by expression of GFP-MCOLN1 was greatly reduced when the ALG-2-binding domain was mutated (44-RLK/AAA). GST-MCOLN1-NTail pulled down not only ALG-2 but also Sec31A and Sec13 in similar quantities in EGTA-eluted fractions. Since GST-MCOLN1-NTail did not pull down the Sec31A/Sec13 complex in ALG-2-depleted cells, the interaction between MCOLN1 and the Sec31A/Sec13 complex was thought to be indirect by the adaptor function of ALG-2 [45]. Because of the differences between working places of Sec31A/Sec13 (ER-to-Golgi transport) and MCOLN1 (endolysosome pathway), interaction of MCOLN1 with Sec31A/Sec13 in the presence of Ca^2+^/ALG-2 on the ER membrane seems unlikely. Those authors used only a GST-MCOLN1-NTail pulldown assay and analyzed fractions eluted with 10 mM EGTA. Unknown ALG-2-binding proteins, capable of interacting with MCOLN1-NTail but not eluted from the beads, cannot be excluded as genuine ALG-2-interacting proteins.

The mammalian target of rapamycin (mTOR) kinase phosphorylates TRPML1 (MCOLN1) and inactivates the Ca^2+^-efflux channel TRPML1 of the lysosome [100]. Interestingly, Sec13 was found to be one of subunits constituting the GATOR2 (GTPase-activating proteins toward Rags 2) complex that indirectly regulated mTOR complex 1 (mTORC1) [101]. Since Sec31A was not contained in the GATOR2 complex, the available Sec31A and Sec13 by the GST-MCOLN1-NTail pulldown assay must have been a cytosolic free Sec31A/Sec13 heterodimer. A recent study has shown that lysosomal TRPML1 functions as a ROS sensor [102]. Release of Ca^2+^ from the lysosome triggers calcineurin (Ca^2+^/calmodulin-activated Ser/Thr phosphatase)-dependent nuclear translocation of transcription factor EB (TFEB), which regulates autophagy and lysosome biogenesis [102]. Potential involvement of ALG-2 in the endolysosome system would expand roles of ALG-2 in organelle-associated Ca^2+^-signaling other than the ER.

#### 6.3.3. PLSCR3

Phospholipid scramblases (PLSCRs) are palmitoylated membrane-anchored proteins and have Ca^2+^-dependent phospholipid scrambling activities in vitro, but phosphatidylserine (PtdSer) externalization activity in vivo has been skeptically argued [103,104]. Plasma membrane PtdSer externalization for “Eat-me signal” during apoptosis is catalyzed by other factors, i.e., TMEM16F and Xkr8 [105]. PLSCR3 promotes translocation of cardiolipin from the mitochondrial inner membrane to the outer membrane [106]. This process is coupled with a regulatory role of PLSCR3 in cardiolipin de novo biosynthesis and its resynthesis [107]. Knockdown of PLSCR3 decreased the delivery of pro-mitophagy-stimulated mitochondria to autophagosomes, which was mediated by binding of the autophagy protein LC3 to cardiolipin [108]. *PLSCR3* gene knockout (*Plscr3*^−/−^) mice accumulated abdominal fat and displayed insulin resistance and glucose intolerance [109]. On the other hand, overexpression of PLSCR3 inhibited the adipogenesis of mouse 3T3-L1 cells by suppressing induction of the mRNAs of late stage pro-adipogenic transcription factors [110]. The biological significance of ALG-2 binding to the N-terminal PRR of PLSCR3 has not been clarified. PLSCR3 was secreted to the extracellular space by an unconventional pathway, most likely by exosomes, the secretion of which was significantly suppressed by expression of a dominant negative ATPase-defective form of VPS4B [111]. ALG-2 may bridge PLSCR3 and other proteins involved in exosome secretion.

## 7. Interplay of PEF Proteins

### 7.1. Peflin

PEF protein genes must have been duplicated several times, diverged, and acquired specific functions in vertebrates [14]. Rayl et al. [112] reported that knockdown of peflin enhanced VSV-G transport from the ER to Golgi and that double knockdown of peflin and ALG-2 canceled the knockdown effect of peflin in normal rat kidney (NRK) cells. Their plausible explanation is that knockdown of peflin diminishes the formation of a peflin/ALG-2 heterodimer and causes an increase in ALG-2 homodimer formation, resulting in activation of the PRR of Sec31A for interactions with inner shell components or cargo-associating proteins. Peflin tends to dissociate from ALG-2 in the presence of Ca^2+^ under an immunoprecipitation condition including detergent [18]. However, it remains to be established whether the ALG-2/peflin heterodimer remains and retains the ability to bind target proteins under physiological conditions. Alternatively, the peflin monomer and ALG-2/peflin heterodimer might have specificities different from that of the ALG-2/ALG-2 homodimer for target binding. It is also possible that an ALG-2/peflin heterodimer bridges partners that are different from those bridged by an ALG-2/ALG-2 homodimer.

### 7.2. Sorcin

Sorcin is classified into the group II PEF subfamily including typical calpains due to the similarity of the positions of intron insertions in the genome as well as amino acid sequence similarity in the EF1 region [14]. However, the entire sequence of sorcin is more similar to those of group I PEF proteins including ALG-2 and peflin [113]. Sorcin interacts with a variety of proteins including ryanodine receptor (RyR), sarcoplasmic/endoplasmic reticulum Ca^2+^-ATPase (SERCA), Polo-like kinase 1 (PLK1), and carbohydrate-responsive element-binding protein (ChREBP) (see Refs. [114,115] and references therein). Interestingly, ALG-2 and sorcin share AnxA7 (synexin) and AnxA11 as common interacting proteins [42,43,116]. Sorcin was recruited to the chromaffin granule membrane by AnxA7 in a Ca^2+^-dependent manner and inhibited AnxA7-mediated chromaffin granule aggregation [116]. Both AnxA11 and sorcin localized to the midbody during cytokinesis, and knockdown of AnxA11 or sorcin caused failure in completing cytokinesis and induced cell death [115,117]. Cell cycle abnormality was also reported for ALG-2 knockdown cells [118]. Sorcin associated with ALG-2, most probably by binding to the Ala/Gly/Pro-rich N-terminal tail of ALG-2, in the presence and absence of Ca^2+^ in in vitro binding assays using a surface plasmon resonance (SPR) biosensor [56]. Relatively large estimated dissociation constants (*Kd* = 3.5~12 μM) suggest that the interaction is weak and that binding of the two different PEF proteins occurs under limited conditions in restricted areas where both proteins accumulate in the cells. Immunofluorescence microscopic analysis showed that sorcin, AnxA7, and AnxA11 as well as ALG-2 partly colocalized at the midbody [56,115,117]. Since sorcin does not bind ALIX [3], localization of sorcin to the midbody may depend largely on binding to AnxA11, which is known to be required for midbody formation [117]. Interplay of sorcin, AnxA11, and ALG-2 might contribute to the regulation of PLK1, which phosphorylates sorcin [115] and ALIX to induce an open conformation for initiation of ESCRT-III assembly [67].

### 7.3. Calpains

Calpains are nonlysosomal intracellular Ca^2+^-activated cysteine proteases and are classified into two major groups: typical calpains (containing PEF domains) and atypical calpains (lacking PEF domains) [22]. Among the typical calpains, conventional m- or μ-calpains, which have a common small subunit also containing a PEF domain, are required for Ca^2+^-facilitated survival after plasma membrane damage [119]. Interplay of acute membrane sealing of the injury sites by activation of the ALG-2/ALIX/ESCRT-III pathway and localized remodeling of the cortical cytoskeleton by proteolytic calpain actions may be necessary for full recovery of injured cells [83,84,85]. Calpain-7, an atypical calpain lacking the PEF domain, possesses a tandem repeat of MIT (microtubule-interacting and transport) domains [113]. Budding yeast lacks typical calpains but retains Rim13p, which associates with Snf7p (CHMP4) and is recruited to the endosomal membrane by ESCRTs [120]. The yeast calpain cleaves Rim101p (a transcription factor containing a YP*X*L/I motif), which interacts with Rim20p (an ALIX homolog, a paralog of yeast Bro1p) to induce gene expression for ambient pH adaptation [120]. Physiological substrates of mammalian calpain-7 have not been determined yet, but proteolytic activity of calpain-7 is significantly enhanced by ESCRT proteins including IST1, with which calpain-7 directly interacts through the MIT domains [121,122]. Calpain-7 is involved in proteolytic downregulation of internalized EGFR in the endosomal pathway [123]. Although the roles of ALG-2 in calpain-7 functions remain to be clarified, the physical link of IST1/ALG-2 and IST1/calpain-7 suggests acquisition of diversity in vertebrates by creating new genes for typical calpains (working ESCRT-independently) by fusion of the genetically separated prototypic calpain catalytic domain and the PEF domain [113]. In mammalian cells, connections of CHMP4, ALIX (and HD-PTP), ALG-2, IST1, and calpain-7 (Figure 6) may have specific proteolytic missions, which need to be clarified.

## 8. Interaction of ALG-2 with Membrane Receptors and Signal Transducers

ALG-2 is associated with cancer development [5,124], and clinical investigation of cancer cells and tissues have revealed the usefulness of monitoring the expression of ALG-2 (often quoted PDCD6) as a biomarker for prognosis [125,126,127]. ALG-2 and ALIX interact with pro-caspase-8 and tumor necrosis factor (TNF) α receptor-1 (TNF-R1), respectively, in the death-inducing signaling complex [48]. The ALIX/ALG-2 complex is thought to allow recruitment of pro-caspase 8 onto endosomes containing TNF-R1. Park et al. [128] reported that ALG-2 promoted TNFα-dependent apoptosis through the activation of NF-κB signaling pathways, changing the expression of pro-apoptotic and anti-apoptotic factors. ALG-2 associates with endothelial growth factor receptor 2 (VEGFR2) and suppresses the PI3K/mTOR/p70S6K signaling pathway [49]. ALG-2 has been reported to interact with cancer- or apoptosis-associated Ser/Thr-kinases such as Raf-1 [50] and DAPK1 (death-associated protein kinase 1) [51] in a Ca^2+^-dependent manner but with ASK1 Ca^2+^-independently [52]. 

## 9. Association with the Nucleus

The nuclear function of ALG-2 is also becoming apparent from findings of interactions of ALG-2 with RNA-associated proteins CHERP (Ser/Arg-rich splicing factor superfamily) and RBM22 (RNA-binding motif 22, spliceosomal nuclear protein), which contribute to the regulation of alternative splicing of pre-mRNAs [53,55,129]. PATL1, a cytoplasmic P-body mRNA decay factor, also co-localizes at subnuclear regions with Ser/Arg-rich splicing factor SC35 (SRSF2) and is thought to be involved in nuclear RNA processing [130]. Nuclear Ca^2+^ concentration is regulated partly by cytosolic Ca^2+^ passing through the nuclear pore complex (NPC) and also partly independently by release of ER-nuclear envelope (NE)-stored Ca^2+^ by ion channels localized on the nuclear membrane facing the nucleoplasm [131]. The ESCRT-III/VPS4 machinery functions for surveillance of NPC integrity and resealing of the reformed NE during mitosis (see Ref. [11] for review). When immune cells and tumor cells migrate through the tight interstitial space, extensive deformation of the cell and its nucleus causes membrane injury. The ESCRT-III/VPS4 machinery has been shown to play critical roles in the repair of this interphase NE bleb rupture [132,133]. It remains to be established how the ESCRT-III/VPS4 machinery is recruited to the wounded sites. AnxA11 has been reported to associate with the reforming nuclear envelope in late telophase of mitosis [134]. Translocation of AnxA11 to wounded NE sites in interphase might activate a pathway similar to the ALG-2/ALIX/ESCRT pathway, which has been shown to be involved in the plasma membrane repair [84,85].

## 10. Perspective

As described in this review, various ALG-2-interacting proteins have been identified, but roles of ALG-2 on a molecular basis have been partially clarified only in limited cases of the ALIX-associated ESCRT system and in the COPII system. In both cases, membranes provide assembly sites of the multiprotein complex, and extracellular or organelle-stored Ca^2+^ serves as a source for activation of ALG-2. The ESCRT system and the COPII system are conserved in eukaryotes. ALG-2 homologs in lower eukaryotes may play similar roles but in a different mode of action in each organism. Yeast and invertebrate homologs of human ALIX and Sec31A also have PRRs, but sequences similar to those of the ALG-2-binding motifs shown in Figure 3 have not been found. The budding yeast ALG-2 homolog named Pef1p binds yeast Sec31p at the PRR in the absence of Ca^2+^, but not in the presence of Ca^2+^, in a binding assay mixture [135]. 

Calmodulin binds many target proteins by recognizing different motifs in Ca^2+^-dependent and Ca^2+^-independent manners and displays flexible structures for binding [136,137]. Calmodulin also bridges proteins with the two lobes (the N-terminal and the C-terminal EF-hand pairs) inter- and intra-molecularly by binding to two target motifs [138,139]. It remains to be established whether one monomeric molecule of ALG-2 has a capacity to bridge two binding partners or only a homodimer can bridge them due to steric restrictions. In addition to the three hydrophobic pockets (Pockets 1, 2, and 3) in ALG-2 (Figure 2), computational algorithms predict one more hydrophobic cavity near the interface of the two dimer molecules [140]. It would be intriguing to see whether Pocket 4 accepts a new type of motif that has not been determined yet. In conclusion, ALG-2 interacts with a variety of proteins with diverse modes of recognition and may play roles as a Ca^2+^-dependent adaptor to relocate target proteins to the proper subcellular space, such as to the plasma and organelle membranes and to the nucleus. Identification of novel interacting proteins and elucidation of functional roles should provide more lines of evidence that dimeric ALG-2 bridges proteins to expand the interacting network and contributes to regulation of Ca^2+^-dependent membrane trafficking. 

## Figures and Tables

**Table 1:** ALG-2-interacting proteins reported in mammals. This table presents a list of ALG-2-interacting proteins published. For convenience, UniProt IDs for human proteins are indicated. Underlined numeric characters of binding motifs indicate that a minimal sufficient binding capacity was confirmed by in vitro binding assays. Full protein names of interacting partners: ALIX, ALG-2-interacting protein X; ASK1, apoptosis signal-regulating kinase 1; CHERP; calcium homeostasis and endoplasmic reticulum protein; DAPK1, death-associated protein kinase 1; HD-PTP, His domain-containing protein tyrosine phosphatase; IST1, increased sodium tolerance-1; PATL1, protein associated with topoisomerase II (PAT)-like 1; PLSCR3, phospholipid scramblase 3; RBM22, RNA-binding motif protein 22; TSG101, tumor susceptibility gene 101; VEGFR2; vascular endothelial growth factor receptor 2; VPS, vacuolar protein sorting. Abbreviated names of employed methods: Y2H, yeast two-hybrid; IP, immunoprecipitation; PD, pulldown; FW, Far Western; SPR, surface plasmon resonance. PRR, Pro-rich regions; PEF, penta-EF-hand. Notes: nd, not determined; n/a, not applicable.

Protein Name	Alternative Name	UniProt ID (Human)	Binding Domain/Region	Binding Motif	Binding to ΔGF122	Method	Function System	Reference
ALIX	AIP1, PDCD6IP	Q8WUM4	PRR	1, 3-like	No	Y2H, IP, PD, FW, SPR	ESCRT accessory	[2,3,25,28,30,31]
HD-PTP	PTPN23	Q9H3S7	PRR	1-like	nd	Y2H, PD, FW	ESCRT accessory	[32,54]
TSG101	-	Q99816	PRR	1-like	No	Y2H, IP, PD, FW	ESCRT-I	[33,34,54]
VPS37B	-	Q9H9H4	PRR	1-like	No	IP, PD, FW	ESCRT-I	[35]
VPS37C	-	A5D8V6	PRR	1-like	nd	IP, PD, FW	ESCRT-I	[35,54]
IST1	-	P53990	PRR	3	No	PD, FW	ESCRT-III	[36]
Sec31A	-	O94979	PRR	2	Yes	IP, PD, FW	COPII outer shell	[37,38,39,40,54]
PLSCR3	PLS3, Scr3	Q9NRY6	PRR	1, 2	Yes	IP, PD, FW, SPR	cardiolipin translocation	[41,54]
annexin A11	AnxA11	P50995	PRR	1-like	No	Y2H, PD, FW, SPR	phospholipid binding	[41,42,43,54]
annexin A7	AnxA7, synexin	P20073	PRR	1-like	No	PD, FW, SPR	phospholipid binding	[41,43,54]
copine-4	CPNE4	Q96A23	VWFA	nd	nd	Y2H, PD	phospholipid binding	[44]
Mucolipin-1	MCOLN1, TRPML1	Q9GZU1	N-tail	ABH	Yes/No	PD	ion channel	[45]
Scotin	SHISA5	Q8N114	PRR	1-like	No	PD, IP, FW	apoptosis	[46,54]
Fas *^1^	APO-1, CD95	P25445	nd	nd	nd	Y2H, IP, PD	apoptosis	[47]
pro-caspase 8	-	Q14790	nd	nd	nd	IP	apoptosis	[48]
VEGFR2	FLK1, KDR	P35968	801–1180	nd	nd	Y2H, IP	RTK, angiogenesis	[49]
Raf-1	RAF1	P04049	nd	nd	nd	Y2H, IP	Ser/Thr kinase	[50]
DAPK1	-	P53355	nd	nd	nd	Y2H, IP	Ser/Thr kinase	[51]
ASK1 *^2^	MAP3K5	Q99683	941–1375	nd	No	PD, IP	Ser/Thr kinase	[52]
RBM22	ZC3H16	Q9NW64	PRR	2-like	n/a *^3^	Y2H, FW	pre-mRNA splicing	[53,54]
PATL1	Pat1b	Q86TB9	PRR	2	Yes	PD, IP, FW	RNA processing	[54]
CHERP	SCAF6	Q8IWX8	PRR	1, 2-like	nd	IP, FW	pre-mRNA splicing	[54,55]
ALG-2	PDCD6	O75340	EF5	nd	nd	Y2H, IP	PEF family	[3,17]
peflin	PEF1	Q9UBV8	EF5	nd	nd	Y2H, IP	PEF family	[18]
sorcin *^4^	-	P30626	PEF	nd	nd	SPR	PEF family	[56]

*^1^ Interaction is controversial and probably incorrect (see Ref. [57] for critical evaluation); *^2^ interaction was observed in the presence of Ca^2+^ but also at a similar strength in the presence of 5 mM EGTA [52]; *^3^ interaction was negative by the GST-ALG-2 pulldown assay, probably due to masking of binding sites [54]; *^4^ the N-terminal APG-rich region of ALG-2 binds sorcin in the presence and absence of Ca^2+^ [56].

## Abbreviations

ABM	ALG-2-binding motif
ALIX	ALG-2-interacting protein X
AnxA11	annexin A11
CEP55	centrosomal protein of 55 kDa
CIE	clathrin-independent endocytosis
CME	clathrin-mediated endocytosis
COPII	coat protein complex II
CTxB	cholera toxin B
EGFR	epidermal growth factor receptor
ER	endoplasmic reticulum
ERES	ER exit site
ESCRT	endosomal sorting complex required for transport
GST	glutathione *S*-transferase
ILV	intraluminal vesicle
LBPA	lysobisphosphatidic acid
mTOR	mammalian target of rapamycin
MIT	microtubule-interacting and transport
MVB	multivesicular body
NE	nuclear envelope
NPC	nuclear pore complex
PEF	penta-EF-hand
PRR	Pro-rich region
TNFα	Tumor necrosis factor alpha
VSV-G	vesicular stomatitis virus envelope glycoprotein
